# Neural Network Model for Predicting Student Failure in the Academic Leveling Course of Escuela Politécnica Nacional

**DOI:** 10.3389/fpsyg.2020.515531

**Published:** 2020-12-09

**Authors:** Iván Sandoval-Palis, David Naranjo, Raquel Gilar-Corbi, Teresa Pozo-Rico

**Affiliations:** ^1^Departamento de Formación Básica, Escuela Politécnica Nacional, Quito, Ecuador; ^2^Department of Developmental Psychology and Didactics, University of Alicante, Alicante, Spain

**Keywords:** neural network, predictive modeling, student success, academic leveling course, learning analytics, academic performance

## Abstract

The purpose of this study is to train an artificial neural network model for predicting student failure in the academic leveling course of the Escuela Politécnica Nacional of Ecuador, based on academic and socioeconomic information. For this, 1308 higher education students participated, 69.0% of whom failed the academic leveling course; besides, 93.7% of the students self-identified as mestizo, 83.9% came from the province of Pichincha, and 92.4% belonged to general population. As a first approximation, a neural network model was trained with twelve variables containing students’ academic and socioeconomic information. Then, a dimensionality reduction process was performed from which a new neural network was modeled. This dimension reduced model was trained with the variables application score, vulnerability index, regime, gender, and population segment, which were the five variables that explained more than 80% of the first model. The classification accuracy of the dimension reduced model was 0.745, while precision and recall were 0.883 and 0.778, respectively. The area under ROC curve was 0.791. This model could be used as a guide to lead intervention policies so that the failure rate in the academic leveling course would decrease.

## Introduction

The concern of universities about the quality of the educational service they offer has triggered several and continuous evaluation processes to detect the underlying problems and act in this regard (Sandoval et al., 2019). The problems identified through these evaluation processes include several aspects of the education system; nevertheless, one of the most serious is the high rate of student failure in university education, which is significantly higher during the first year of studies. For instance, in South America during the last decade, about 50% of students failed their university studies during their first year (Parrino, 2010). The results of several studies provide evidence that student failure is influenced by an interaction of various factors which are decisive throughout the academic process (Amaya et al., 2015; Montoya Gutiérerz, 2016; Amo and Santelices, 2017; Lara et al., 2017).

In Ecuador, several studies have been performed to identify factors influencing student failure. In this context, factors such as family monthly income, type of school, type of housing, and even gender have been identified as components that intervene in the phenomenon of student failure (Sandoval et al., 2018). At the same time, the government and universities have proposed affirmative action policies to help students overcome the difficulties triggered by the influence of these factors. Therefore, identifying these factors and analyzing their influence on students’ academic performance is an important process to be performed in order to early identify at-risk students and, consequently, implement corrective actions in the educational process (Di Caudo, 2015; Sandoval et al., 2019).

Artificial neural networks (ANNs) have optimal features to conduct this type of analysis due to its excellent prediction and classification performance. An artificial neural network is a reticular computer system that learns from experience by self-modifying its connections; in this way, data prediction can be estimated based on a wide range of information (Cao et al., 2018; Zhou et al., 2018; Bouwmans et al., 2019).

Artificial neural networks are helpful tools used for data analysis with which functional relationships between variables can be found and modeled. Indeed, they allow exploring relationships or models that otherwise could not be found, for example, by using traditional statistical procedures (Bouwmans et al., 2019). In addition, due to the fact that they are a type of machine learning algorithm, ANNs have advantages over traditional statistical methods when applied to studies in which input data are incomplete or ambiguous by nature. They also have good performance when studying non-linear problems or data with a lot of “noise” and can be applied even without meeting theoretical assumptions related to traditional statistic because ANNs decode the information implicit in the data (Cao et al., 2018).

Currently, ANNs are widely applied to solve prediction and classification problems in areas as diverse as Meteorology and Spectroscopy (Timoshenko et al., 2018; Lee and Kim, 2019). The application of ANN for studying academic performance has gained significance in recent years not only because of its higher performance but also because of the findings regarding factors that influence the educational process (Vandamme et al., 2007; Marbouti et al., 2016; Baars et al., 2017; Mason et al., 2018; Figueiredo et al., 2019).

The purpose of this study was to develop a neural network model for predicting student failure in the academic leveling course of the Escuela Politécnica Nacional of Ecuador based on academic and socioeconomic information.

## Methods

### Participants

The participants in this study were 1308 higher-education students from the Escuela Politécnica Nacional of Ecuador whose characteristics are shown in Table 1.

**TABLE 1:** Characteristics of the study population.

Variable	Distribution			
Gender	63.2% male			
	36.8% female			
Ethnicity	93.7% mestizo	0.9% Mulatto		
	2.7% indigenous	0.3% black		
	1.1% white	0.1% Montubio		
	1.0% afro-descendant	0.2% other		
Province of origin	83.9% Pichincha	1.2% Carchi	0.5% Chimborazo	0.1% Santa Elena
	3.1% Tungurahua	1.1% Imbabura	0.5% Sucumbíos	0.1% Zamora Chinchipe
	2.8% Cotopaxi	1.0% El Oro	0.3% Guayas	0.1% Orellana
	2.4% Santo Domingo de los Tsáchilas	0.5% Azuay	0.3% Manabí	
	1.4% Esmeraldas	0.5% Bolívar	0.2% Loja	
Population segment	92.4% general population			
	5.6% affirmative action			
	1.5% territorial merit			
	0.5% high-performance group			
Regime	35.9% Sierra			
	64.1% Costa			
Academic performance	31.0% passed			
	69.0% failed			

### Measures

The variables to predict (levpass) states whether a student failed the academic leveling course while the following twelve variables were considered as predictors:

1. Application score (appscore): The score achieved by the students in the university application exam. This exam is graded between 400 and 1000 points. The higher the score, the higher the student’s performance in the exam. The application score does not consider students’ high school GPA.
2. Vulnerability index (vulnind): This index shows the relative socioeconomic vulnerability of a student. It is rated over 1000 points. The higher the index, the lower the socioeconomic vulnerability. The index is calculated from the information stated by the students in a socioeconomic survey during the university application process.
3. Gender (gender): student’s gender.
4. Population segment (popsgmnt): It is a way of classifying students according to their academic performance and socioeconomic characteristics. There are four types of population segments:
  • High-performance group (HPG): This group is formed by the applicants best scored in the university application exam.
  • Territorial merit: This group is formed by the best graduates of the educational institutions whether these are public, municipal, or fisco-misional schools.
  • Affirmative action: This group is formed by the applicants in a situation of vulnerability which considers their socioeconomic situation, disability, territoriality (provinces with a lower rate of access to higher education), and other conditions of vulnerability.
  • General population: This group is formed by the students who do not belong to any of the other population segments.
5. Application priority (appprior): The priority of the chosen degree made by the student during the university application process.
6. Application instance (appinstance): The instance in which a student applied to the university. It can be the first, the second, or the third instance.
7. Assignation instance (assiginstance): The instance in which the student was assigned a place in the university. It can be the first, the second, the third, the fourth, or the fifth instance of assignment of places.
8. School type (schooltype): The type of school a student comes from. It can be public, municipal, private, lay private, lay, religious private, fisco-misional, or foreign.
9. Regime (regime): The academic regime in which a student enters the university. It can be Costa or Sierra. The Sierra regime is analogous to the autumn semester, which runs from September to February, whereas the Costa regime is analogous to the spring semester, which runs from March to August.
10. Province (province): the province a student comes from.
11. Ethnicity (ethnicity): The ethnic group to which a student belongs. This information is self-stated by each student.
12. Disability (disability): This variable states whether a student has a disability.

### Procedures

Data were collected from the existing computer records in the administration of the Escuela Politécnica Nacional of Ecuador with permission granted by the academic staff of the Institution. The data provided by the institution were anonymous.

### Artificial Neural Network Modeling

In this study, an ANN model was used to predict student failure from their academic and socioeconomic information.

First, data were partitioned into training (70% of cases), validation (15% of cases), and testing (15% of cases) sets by a random sampling process. Then, an ANN model was trained on the training data set. The hyperparameters of the ANN model were one hidden layer, logistic activation function, Adam optimization algorithm, learning rate of 0.0001, and 4000 as the maximum number of iterations. Different ANN models with varying numbers of neurons in the hidden layer were validated. The number of neurons in the hidden layer was defined according to Eq. (1):

(1) $$\frac{2}{3}NIL+NOL\leq NHL\leq 2NIL\;$$

where NHL is the number of neurons in the hidden layer, NIL is the number of neurons in the input layer, and NOL is the number of neurons in the output layer.

Once the model reached the maximum fit on the validation set, the ANN model was tested on the testing set in order to measure its performance (Teoh et al., 2006).

### Dimensionality Reduction

As a first approximation, the ANN model was trained with the twelve variables containing students’ academic and socioeconomic information which resulted in a complex model. Therefore, in order to reduce the dimensions of the model, three tests were performed: Garson test, ANOVA, and chi-squared test. From Garson test, the relative importance of each of the variables considered in the model was determined, whereas, from ANOVA, it was determined whether there was a significant difference in the application score and the vulnerability index between the students who failed and those who passed the leveling course. On the other hand, the chi-squared test was performed to determine whether categorical variables were independent of each other. For ANOVA and chi-squared test, a significance level of *p* = 0.05 was chosen. Consequently, only those variables that had a relative importance greater than 5% and with a *p*-value less than 5% were chosen to train a new model. This dimension reduced model was trained according to the criteria stated in the preceding section (Vandamme et al., 2007; Helal et al., 2018).

### Model Performance Evaluation Criteria

Although the procedure used to train the models allows obtaining a first approximation of their performance, this procedure is very susceptible to problems of overfitting on the training and validation sets. Therefore, the general performance of the models was determined by cross-validation with *k* = 10, which minimizes the effects of overfitting and selection bias (Cawley and Talbot, 2010; Juba and Le, 2019). The performance of the model was evaluated through the following indicators:

• Accuracy: the ratio of the total number of correct predictions to the total number of predictions.
• Precision: the ratio of positive class predictions that actually belong to the positive class.
• Recall: the ratio of positive class predictions made out of all positive cases.
• Area under the receiver operating characteristic (ROC) curve, which provides an aggregate measure of performance across all possible classification thresholds.

Data analysis and modeling were performed in SPSS 22, Orange 3.22.0, and RStudio Version 1.2.1335.

## Results

**FIGURE 1:**
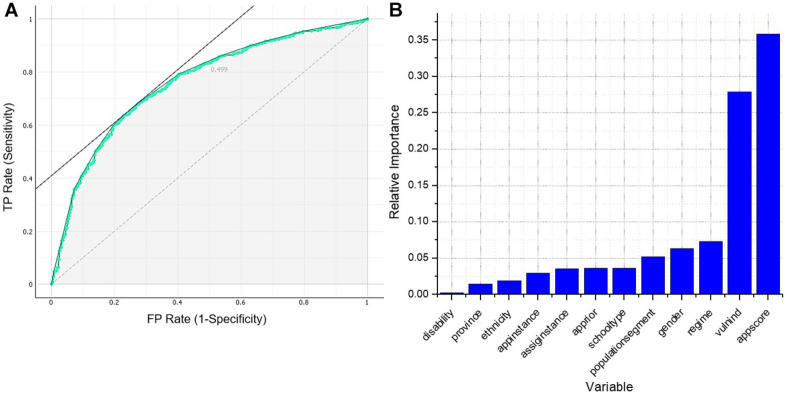
**(A)** ROC curve of neural network 48–39–1. **(B)** Garson’s weighted importance of the variables of neural network 48–39–1.

The ANN trained with the twelve predictor variables was modeled on an architecture of forty-eight neurons in the input layer and one neuron in the output layer. Each of the neurons in the input layer corresponds to each of the possible categories in the predictor variables. The highest performance for this model was achieved with thirty-nine neurons in the hidden layer; hence, the resulting architecture of this ANN can be written as 48–39–1. The classification accuracy for this model was 0.732, while precision and recall were 0.833 and 0.789, respectively. Figure 1A shows the ROC curve; the resulting value for the area under this curve was 0.757.

The results of the Garson test are presented in Figure 1B. Application score, vulnerability index, regime, gender, and population segment were the five variables that showed a relative importance greater than 5%, where the application score and vulnerability index resulted to be the most important for the model. Also, these variables explained more than 80% of the model.

Chi-squared test and ANOVA’s *p*-values are presented in Table 2. The application score and the vulnerability index of the students who passed the leveling course were significantly different from those who failed. Also, it is noted that disability, province, and ethnicity are not only variables with less relative importance according to the Garson test but also independent of student failure according to the chi-squared test. Additionally, regime is not independent of application priority, application instance, assignation instance, school type, and province, then, when including regime in the new model, the effect of the aforementioned five variables will be considered indirectly.

**TABLE 2:** Chi-squared test and ANOVA’s *p*-values of the twelve predictor variables.

Variable	Levpass	Gender	Popsgmnt	Regime	Ethnicity	Apprior	Appinstance	Assiginstance	Schooltype	Province	Disability
Appscore	0.000										
Vulnind	0.001										
Levpass	–										
Gender	0.007	–									
Popsgmnt	0.034	0.377	–								
Regime	0.000	0.136	0.000	–							
Ethnicity	0.144	0.063	0.005	0.037	–						
Apprior	0.000	0.469	0.198	0.000	0.587	–					
Appinstance	0.001	0.018	0.005	0.006	1.000	0.001	–				
Assiginstance	0.000	0.030	0.028	0.000	0.991	0.000	0.000	–			
Schooltype	0.001	0.026	0.596	0.000	0.000	0.156	0.001	0.017	–		
Province	0.089	0.195	0.000	0.000	0.007	0.958	0.976	1.000	0.000	–	
Disability	0.180	0.127	0.403	0.000	0.353	0.043	0.649	0.103	0.027	1.000	–

According to the results presented above, a dimension reduced model was trained with the variables application score, vulnerability index, regime, gender, and population segment.

**FIGURE 2:**
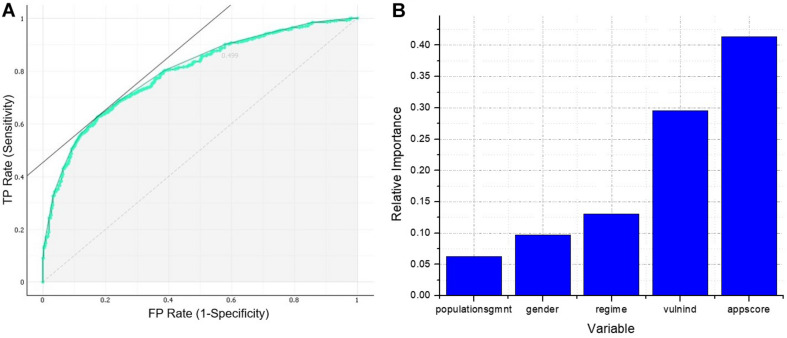
**(A)** ROC curve of neural network 7–4–1. **(B)** Garson’s weighted importance of the variables of neural network 7–4–1.

The dimension reduced ANN model had seven neurons in the input layer and one neuron in the output layer. The highest performance for this model was achieved with four neurons in the hidden layer, which resulted in a 7–4–1 architecture. The classification accuracy for this model was 0.745, while precision and recall were 0.883 and 0.778, respectively. Also, the area under the ROC curve, shown in Figure 2A, was 0.791. The dimension reduced model was not only far simpler than the initial one but also higher on classification performance.

The results of the Garson test for the dimension reduced model are presented in Figure 2B, which indicates that all the five chosen variables had a relative importance greater than 5%, and, again, the two most important were application score and vulnerability index.

## Discussion

The purpose of this study was to identify as early and reliable as possible students that might fail the academic leveling course. Even though both models did not achieve a classification accuracy higher than 0.800, the dimension reduced model could be used as a guide to lead intervention policies so that the high rates of failure in the academic leveling course would decrease (Marbouti et al., 2016).

There are multiple reasons why both models could not classify correctly failing students. First, from a theoretical perspective, it is not possible to accurately model student failure because more factors could influence this phenomenon and it is almost impossible to include them all in the model. Therefore, any model will have limitations, resulting in misclassifying problems (Vandamme et al., 2007; Marbouti et al., 2016). Another reason is that biased and unbalanced data can significantly influence the classification performance of a model. However, for machine learning algorithms, omitting variables that have bias and balancing data should be performed cautiously (Cawley and Talbot, 2010). For instance, in this study, the variables ethnicity, province, and population segment have a considerable bias since 93.7% of the students self-identifies as mestizo, 83.9% comes from Pichincha, and 92.4% belongs to general population. Nevertheless, although ethnicity and province were not considered in the dimension reduced model, population segment was actually included to train this model. This is because, according to historical information from the Escuela Politécnica Nacional, students of affirmative action are more likely to fail the leveling course in comparison to students from other population segments. Thus, omitting variables that have bias and balancing data should only be done to decrease the bias in the sample data and make them more closely represent the real population. In fact, if population segment had not been included in the model, the omitted-variable bias would have occurred (Cawley and Talbot, 2010).

On the other hand, the variables that describe students’ behaviors were not considered in this study. This information is of utmost importance since aspects such as previous motivation and the attitude with which students face their studies might be decisive when defining student success. However, variables that describe behavior are very likely to vary over time, so the model might present different results depending on the stage in which it is used (Marbouti et al., 2016; Helal et al., 2018; Mason et al., 2018). This last statement raises the question of whether an early diagnosis of student failure is effective.

Identifying students at risk of failing as early as possible is crucial. Nevertheless, there is important information that is generated as the semester elapses. Student academic performance during the semester might be the best indicator of student success, but predictions based only on this information could provide results when there was not enough time to help students in need of academic support. Furthermore, predictions made too early are likely to be somewhat inaccurate (Helal et al., 2018; Yang and Li, 2018). Thus, in future research, finding the optimal time to utilize a prediction model should be an important point to focus on. Besides, this whole process should be kept as simple as possible so that time and resources would be optimized, and that is one of the most important reasons why the dimension reduced model turns out to be more attractive in terms of applicability (Mason et al., 2018).

The variables considered in the dimension reduced model not only describe socioeconomic as well as academic factors but also confirm their historical influence on student failure. Thus, for example, according to historical data, women have a higher failure rate compared to men, and students with a lower application score and in a situation of vulnerability tend to fail the leveling course. Also, students from the Costa regime are more likely to fail the leveling course in comparison to students from the Sierra regime (Sandoval et al., 2018, 2019). In this context, interventions by the government and the university should aim to mitigate students’ economic difficulties, through financial aid or the strengthening of scholarship programs. On the other hand, regarding academic factors, efforts should focus on offering academic support before the academic leveling course as well as peer tutoring programs during the semester so that student failure and even dropout rates would decrease.

## Conclusion

This study took the first step in identifying factors that influence student failure in the academic leveling course of the Escuela Politécnica Nacional of Ecuador. A dimension reduced artificial neural network was modeled from five variables containing students’ academic and socioeconomic information. The variables used for training this model were application score, vulnerability index, regime, gender, and population segment. The model correctly classified 74.5% of the students that actually failed the leveling course, then, even though the model does not reach the maximum classification performance, it could be used as a guide to lead intervention policies, such as financial aid or academic support, so that the high failure rates in the academic leveling course would decrease.

## Data Availability Statement

The datasets generated for this study are available on request to the corresponding author.

## Ethics Statement

Ethical review and approval was not required for the study on human participants in accordance with the local legislation and institutional requirements. Written informed consent for participation was not required for this study in accordance with the national legislation and the institutional requirements.

## Author Contributions

IS-P was director of the research and carried out the quantitative methods. DN worked on quantitative methods. RG-C and TP-R carried out a theoretical review of the topic. All authors contributed to the article and approved the submitted version.

## Conflict of Interest

The authors declare that the research was conducted in the absence of any commercial or financial relationships that could be construed as a potential conflict of interest.

## References

[B1] Amaya Y. Barrientos E. Heredia D. (2015). Student Dropout Predictive Model Using Data Mining Techniques. *IEEE Latin Am. Trans.* 13 3127–3134. 10.1109/tla.2015.7350068

[B2] Amo C. Santelices M. V. (2017). *Trayectorias universitarias: MÁS QUE PERSISTENCIA O DESERCIÓN. Congresos CLABES.* Panama: Technological University of Panama Available online at: http://revistas.utp.ac.pa/index.php/clabes/article/view/1676/2412

[B3] Baars G. J. A. Stijnen T. Splinter T. A. W. (2017). A Model to Predict Student Failure in the First Year of the Undergraduate Medical Curriculum. *Health Profess. Educ.* 3 5–14. 10.1016/j.hpe.2017.01.001

[B4] Bouwmans T. Javed S. Sultana M. Jung S. K. (2019). Deep neural network concepts for background subtraction: A systematic review and comparative evaluation. *Neur. Net.* 117 8–66. 10.1016/j.neunet.2019.04.024 31129491

[B5] Cao W. Wang X. Ming Z. Gao J. (2018). A review on neural networks with random weights. *Neurocomputing* 275 278–287. 10.1016/j.neucom.2017.08.040

[B6] Cawley G. C. Talbot N. L. C. (2010). On over-fitting in model selection and subsequent selection bias in performance evaluation. *J. Machine Learn. Res.* 11 2079–2107.

[B7] Di Caudo M. (2015). Política de cuotas en Ecuador: me gané una beca para estudiar en la Universidad. *Ponto Vírgula Revista de Ciências Soc.* 1 196–218. 10.47212/tendencias_vii_2019_14

[B8] Figueiredo J. Lopes N. García-Pẽalvo F. J. (2019). Predicting student failure in an introductory programming course with multiple back-propagation. *ACM Int. Conf. Proc. Ser.* 2019 44–49. 10.1145/3362789.3362925

[B9] Helal S. Li J. Liu L. Ebrahimie E. Dawson S. Murray D. J. (2018). Predicting academic performance by considering student heterogeneity. *Knowl. Based Syst.* 161 134–146. 10.1016/j.knosys.2018.07.042

[B10] Juba B. Le H. S. (2019). Precision-Recall versus Accuracy and the Role of Large Data Sets. *Proc. AAAI Conf. Artific. Intel.* 33 4039–4048. 10.1609/aaai.v33i01.33014039

[B11] Lara H. O. Silva J. S. Galeano M. O. Carreño C. C. Ariza A. B. (2017). *Estudio factores asociados a la deserción estudiantil en la universidad minuto de dios de la sede virtual ya distancia. Congresos CLABES.* Available online at: http://revistas.utp.ac.pa/index.php/clabes/article/view/1691 (accessed on Nov 15, 2017).

[B12] Lee D. Kim K. (2019). Recurrent neural network-based hourly prediction of photovoltaic power output using meteorological information. *Energies* 12:215 10.3390/en12020215

[B13] Marbouti F. Diefes-Dux H. A. Madhavan K. (2016). Models for early prediction of at-risk students in a course using standards-based grading. *Comput. Educ.* 103 1–15. 10.1016/j.compedu.2016.09.005

[B14] Mason C. Twomey J. Wright D. Whitman L. (2018). Predicting Engineering Student Attrition Risk Using a Probabilistic Neural Network and Comparing Results with a Backpropagation Neural Network and Logistic Regression. *Res. High. Educ.* 59 382–400. 10.1007/s11162-017-9473-z

[B15] Montoya Gutiérerz G. (2016). *Estudio Factores Asociados Al Abandono Temprano De La Educación Superior. Congresos CLABES.* Available online at: https://revistas.utp.ac.pa/index.php/clabes/article/view/1055 [Nov 3, 2016].

[B16] Parrino C. (2010). *Deserción en el primer año universitario. Dificultades y logros. X Coloquio Internacional Sobre Gestión Universitaria En América Del Sur.* 1–19. https://repositorio.ufsc.br/xmlui/bitstream/handle/123456789/96620/PARRINO.pdf

[B17] Sandoval I. Sánchez T. Naranjo D. Jiménez A. (2019). “Proposal of a mathematics pilot program for engineering students from vulnerable groups of Escuela politécnica Nacional,” in *Proceedings of the LACCEI International Multi-Conference for Engineering, Education and Technology, 2019-July(August)*, (Boca Raton: LACCEI), 10.18687/LACCEI2019.1.1.387

[B18] Sandoval I. Sánchez T. Velasteguí V. Naranjo D. (2018). Factores Asociados Al Abandono En Estudiantes De Grupos Vulnerables. Caso Escuela Politécnica Nacional. *Congr. CLABES* 2018 132–141.

[B19] Teoh E. J. Tan K. C. Xiang C. (2006). Estimating the number of hidden neurons in a feedforward network using the singular value decomposition. *IEEE Trans. Neur. Net.* 17 1623–1629. 10.1109/TNN.2006.880582 17131674

[B20] Timoshenko J. Anspoks A. Cintins A. Kuzmin A. Purans J. Frenkel A. I. (2018). Neural Network Approach for Characterizing Structural Transformations by X-Ray Absorption Fine Structure Spectroscopy. *Phys. Rev. Lett.* 120:225502. 10.1103/PhysRevLett.120.225502 29906159

[B21] Vandamme J. P. Meskens N. Superby−F. (2007). Predicting Academic Performance by Data Mining Methods. *Educ. Econ.* 15 405–419. 10.1080/09645290701409939

[B22] Yang F. Li F. W. B. (2018). Study on student performance estimation, student progress analysis, and student potential prediction based on data mining. *Comput. Educ.* 123 97–108. 10.1016/j.compedu.2018.04.006

[B23] Zhou J. Cui G. Zhang Z. Yang C. Liu Z. Wang L. (2018). Graph Neural Networks: A Review of Methods and Applications. *arXiv* 22:1812.08434.

